# The Competency of Emergency Medicine Residents in Interpreting Hand X-rays Across the Three Major Regions of Saudi Arabia

**DOI:** 10.7759/cureus.59270

**Published:** 2024-04-29

**Authors:** Loui Alsulimani, Basma AlRasheed, Afnan Saeed, Hatim Alabsi

**Affiliations:** 1 Emergency Medicine, King Abdulaziz University Hospital, Jeddah, SAU; 2 Radiology, King Abdulaziz University Hospital, Jeddah, SAU

**Keywords:** viewing devices, training level, assessment, interpretation, saudi arabia, competency, hand x-rays, residents, emergency medicine

## Abstract

Background

Interpreting hand X-rays is crucial for emergency medicine residents to accurately diagnose traumatic injuries and conditions affecting the hand. This study aimed to assess the competency of emergency medicine residents in interpreting hand X-rays across three major regions in Saudi Arabia.

Methodology

We conducted a cross-sectional study involving 100 emergency medicine residents from the Central, Eastern, and Western regions of Saudi Arabia. Participants were presented with 10 clinical case scenarios each accompanied by hand X-rays and were asked to provide their interpretations. Assessment scores were calculated based on the proportion of correct answers for each case.

Results

Half of the participants (50 residents) fell within the age range of 25 to 27 years, with 61 male and 39 female participants, respectively. Residents in the third year of training (R3) exhibited the highest mean score of 74.83% ± 20.46%. Participants using desktops to view the images achieved the highest mean score of 75% ± 10.49% compared to those using smartphones or tablets. Significant associations were found between age (F = 4.072, p = 0.020), training level (F = 3.161, p = 0.028), and choice of viewing device (F = 7.811, p = 0.001) and assessment scores.

Conclusions

Our study highlighted that emergency medicine residents in Saudi Arabia demonstrate competent proficiency in interpreting hand X-rays, with higher competency observed among senior residents (R3 and R4), those aged 28 to 30 years, and those using desktops for image viewing.

## Introduction

Interpreting X-rays is a fundamental skill for emergency medicine (EM) physicians, as it plays a crucial role in diagnosing various traumatic injuries and acute musculoskeletal conditions affecting the patient, making it a crucial part of EM resident program training [1]. The ability to adequately interpret radiological images is essential for ensuring timely and appropriate medical interventions, reducing patient morbidity, and improving overall outcomes [2]. In EM practice, hand X-rays are commonly ordered to assess fractures, dislocations, soft tissue injuries, and degenerative joint diseases, among other conditions. Given the high prevalence of hand injuries and their potential impact on patient function and quality of life, the proficiency of EM residents in interpreting hand X-rays is of paramount importance [1,3-5].

EM residents must possess a comprehensive understanding of normal hand anatomy, including bones, joints, soft tissues, and vasculature, to accurately identify abnormalities and pathology on radiological images [5,6]. Additionally, familiarity with common injury patterns, mechanisms of injury, and clinical presentations is crucial for making accurate diagnoses and providing appropriate management strategies [1,4,7]. While the traditional training methods typically cover basic principles of radiological interpretation, including image acquisition, anatomy recognition, and pathological findings, the translation of theoretical knowledge into clinical practice requires ongoing practice, feedback, and experience [1,8-10]. Several studies have investigated the interpretation competency of residents in various medical specialties, including EM, radiology, orthopedics, and primary care [2-5]. These studies have highlighted variability in interpretation accuracy among residents, influenced by factors such as training level, experience, exposure to specific clinical scenarios, and access to decision-support tools [2,3,5].

In recent years, advancements in technology have transformed the landscape of radiological interpretation, with the widespread adoption of digital imaging systems and picture archiving and communication systems [11]. These digital platforms allow for the rapid acquisition, storage, retrieval, and sharing of radiological images, facilitating remote interpretation and collaboration among healthcare providers [12]. Additionally, the integration of artificial intelligence algorithms and computer-aided detection systems has shown promise in assisting residents with image interpretation, reducing interpretation errors, and improving diagnostic accuracy [13-15]. This study aims to assess the competency level of EM residents in interpreting hand X-rays across three major regions in Saudi Arabia.

## Materials and methods

Ethical considerations

The study received ethical approval from the Research Ethics Committee at the Faculty of Medicine, King Abdulaziz University (reference number: 306-23, cross-sectional/non-interventional). Informed consent was obtained from all participants before their participation in the study. Participants were assured of confidentiality and anonymity, and their participation was voluntary. No identifiable information was collected during the study, and all data were stored securely in compliance with relevant data protection regulations.

Study design

This is a cross-sectional study to assess the competency of EM residents in interpreting hand X-rays across three major regions in Saudi Arabia.

Study participants

The study included 100 EM residents from the Central, Eastern, and Western regions of Saudi Arabia. Participants were selected using convenience sampling methods, with recruitment facilitated through collaboration with residency programs in each region. Inclusion criteria comprised EM residents currently enrolled in training programs, regardless of how long they have been practicing, while exclusion criteria included residents with prior working experience in the radiology department.

Data collection

Data collection was conducted using Google Forms, an online data collection tool provided by Google (Google LLC, Mountain View, CA, USA). The tool presented each participant with 10 clinical cases accompanied by hand X-rays designed to simulate common scenarios encountered in EM practice such as traumatic injuries and musculoskeletal conditions affecting the hand. Participants were instructed to interpret the X-rays and provide their diagnoses for each case. Additionally, participants provided demographic information, including age, gender, training level, residency region, and the device used for viewing the images.

Assessment method

Adequacy was assessed by the number of correct radiological interpretations and missed common injuries. Assessment scores were calculated based on the proportion of correct answers provided by participants for each case. Each correct interpretation was assigned a score of 1, while incorrect interpretations received a score of 0. The total score for each participant was obtained by summing the scores across all 10 cases. To standardize scores and facilitate comparison, the total score was converted to a percentage, with a higher percentage indicating greater competency in interpreting hand X-rays. Mean scores and standard deviations were calculated to summarize interpretation adequacy among participants.

Image selection

The hand X-ray images were selected from Radiopaedia, an international radiology educational web resource, after obtaining permission (Appendices). The cases were selected to represent common emergency conditions commonly encountered in EM practice. The X-ray images were independently interpreted by two fellowship-trained general body radiologists who were not involved in selecting the images and had complete agreement with the diagnoses of all cases. A pilot study was conducted with 10 physicians, with different levels of experience, to assess the clarity of cases and the time required to complete the survey [16].

Data analysis

All data analyses were performed using SPSS software, version 26, developed by IBM Corporation (IBM Corp., Armonk, NY, USA). Analysis of variance was employed to examine differences in mean scores across categorical variables, such as age, gender, training level, residency region, and choice of viewing device. Post-hoc Tukey tests were performed to identify specific group differences when significant main effects were observed. Additionally, independent t-tests were used to compare mean scores between two groups, such as gender or choice of viewing device. Statistical significance was set at p-values <0.05.

## Results

The demographic characteristics of the 100 EM residents included in this study are outlined in Table 1.

**Table 1:** Characters of the included residents (n = 100). R: resident

Parameter		Frequency (%)
Age, year	25 to 27	50 (50%)
	28 to 30	39 (39%)
	31 or more	11 (11%)
Gender	Female	39 (39%)
	Male	61 (61%)
Level of training	R1	32 (32%)
	R2	19 (19%)
	R3	29 (29%)
	R4	20 (20%)
Residency training	Central	34 (34%)
	Eastern	11 (11%)
	Western	55 (55%)
Device used to view the images	Desktop	6 (6%)
	iPad or tablet	4 (4%)
	Smartphone	90 (90%)

Half of the participants, 50 residents, fell within the age range of 25 to 27 years, with 39 (39%) residents aged between 28 and 30 years, and 11 (11%) residents aged 31 years or older. Gender distribution was relatively balanced, with 61 (61%) male and 39 (39%) female participants. Regarding training levels, R1 residents comprised 32 (32%), followed by 29 (29%) R3, 20 (20%) R4, and 19 (19%) R2. Geographically, the study encompassed a diverse representation, with 55 (55%) residents from the Western region, 34 (34%) residents from the Central region, and 11 (11%) residents from the Eastern region. Moreover, 90 (90%) participants, the majority, utilized smartphones as their preferred device for viewing images, while only six (6%) participants used desktops, and four (4%) participants used iPads/tablets. Each participant was supervised by one of the researchers to ensure the selected images were not looked up.

Proportions of correct and incorrect answers provided by the participants across 10 clinical cases accompanied by hand X-rays are presented in Table 2.

**Table 2:** Proportions of correct and incorrect answers for the reviewed cases and X-rays (n = 100). ED: emergency department; DIP: distal interphalangeal joint

Questions	Incorrect answer	Correct answer
1: A 28-year-old female patient presents to the emergency department after falling and sustaining an injury to her right hand (boxer fracture)	39 (39%)	61 (61%)
2: A 10-year-old girl came to the ED complaining of pain in her left hand. According to her mom, she had accidentally closed the door on her left hand (Salter-Harris II)	33 (33%)	67 (67%)
3: A 29-year-old male was involved in a motorcycle accident. Intoxicated polytrauma patient (clinical signs of use of alcohol). Swollen right hand upon physical examination. (Rolando’s fracture + midshaft fracture of the second metacarpal)	59 (59%)	41 (41%)
4: A 25-year-old male patient with right hand pain after a work-related injury. On examination, abrasion and tenderness on the right hand more clearly over the first and second metacarpal bones (Bennett fracture)	60 (60%)	40 (40%)
5: A 30-year-old male who had a motorcycle accident complained of left wrist pain. He had tenderness over his thumb (Scaphoid fracture)	36 (36%)	64 (64%)
6: A 35-year-old male presented with trauma to the right fifth finger one day back. On examination, localized swelling and tenderness in the DIP joint area (Mallet finger)	11 (11%)	89 (89%)
7: A 17-year-old female with a history of acute right-hand trauma after falling down presented with wrist tenderness (Chauffeur fracture)	23 (23%)	77 (77%)
8: A 35-year-old male was hit by metal at work. On examination, tenderness, swelling, and abrasion of the left wrist and hand were noted (Lunate dislocation)	27 (27%)	73 (73%)
9: A 39-year-old male presented after trauma to the left thumb with hyperextension of the thumb. On examination, reduced range of motion and swelling were noted (Gamekeeper’s thumb)	14 (14%)	86 (86%)
10: A 30-year-old male complained of pain in his hand after hitting the edge of his desk today (normal X-ray)	31 (31%)	69 (69%)
Average score, %	Mean‎ ± ‎SD (minimum-maximum)	66.6‎ ± ‎22.1 (10-100)

Notably, varying levels of accuracy were observed across different cases. Responses to cases such as mallet finger accounted for 89 (89%) participants and gamekeeper’s thumb accounted for 86 (86%) participants had higher correct response rates, while cases involving scaphoid fracture had 41 (41%) correct responses and 40 (40%) correct responses for lunate dislocation injury, which garnered lower accuracy levels. The average score across all cases was 66.6%, with a standard deviation of 22.1 and a range of 10 to 100.

Table 3 provides a comprehensive analysis of assessment scores in association with participant characteristics.

**Table 3:** Assessment scores in association with participant characteristics (n = 100). R: resident

Parameter		Assessment test score	Test	P-value
Age, years	25 to 27	60.6‎ ± ‎22.17	F = 4.072	0.020
	28 to 30	73.08‎ ± ‎20.92		
	31 or more	71.82‎ ± ‎19.4		
Gender	Female	68.97‎ ± ‎18.18	t = 0.823	0.413
	Male	65.25‎ ± ‎24.26		
Level of training	R1	58.13‎ ± ‎23.34	F = 3.161	0.028
	R2	66.84‎ ± ‎17.97		
	R3	74.83‎ ± ‎20.46		
	R4	68.5‎ ±‎ 22.31		
Residency training region	Central	67.35 ‎± ‎16.01	F = 0.198	0.820
	Eastern	62.73‎ ±‎ 24.53		
	Western	67.09‎ ± ‎24.92		
Device used to view the images	Desktop	75‎ ± ‎10.49	F = 7.811	0.001
	iPad or tablet	27.5‎ ± ‎17.08		
	Smartphone	67.89 ‎±‎ 21.23		

Significant associations were found between age and assessment scores (F = 4.072, p = 0.020), with participants aged 28 to 30 years demonstrating the highest mean score of 73.08 ± 20.92. Additionally, a significant association was observed across different training levels (F = 3.161, p = 0.028), with residents in R3 training exhibiting the highest mean score of 74.83 ± 20.46. However, no statistically significant correlation was found between gender and assessment scores (t = 0.823, p = 0.413). Interestingly, significant differences in scores were noted based on the device used for viewing X-rays (F = 7.811, p = 0.001), with participants using desktops achieving the highest mean score of 75 ± 10.49. This could be attributed to the images being larger than in handheld devices.

**Figure 1:**
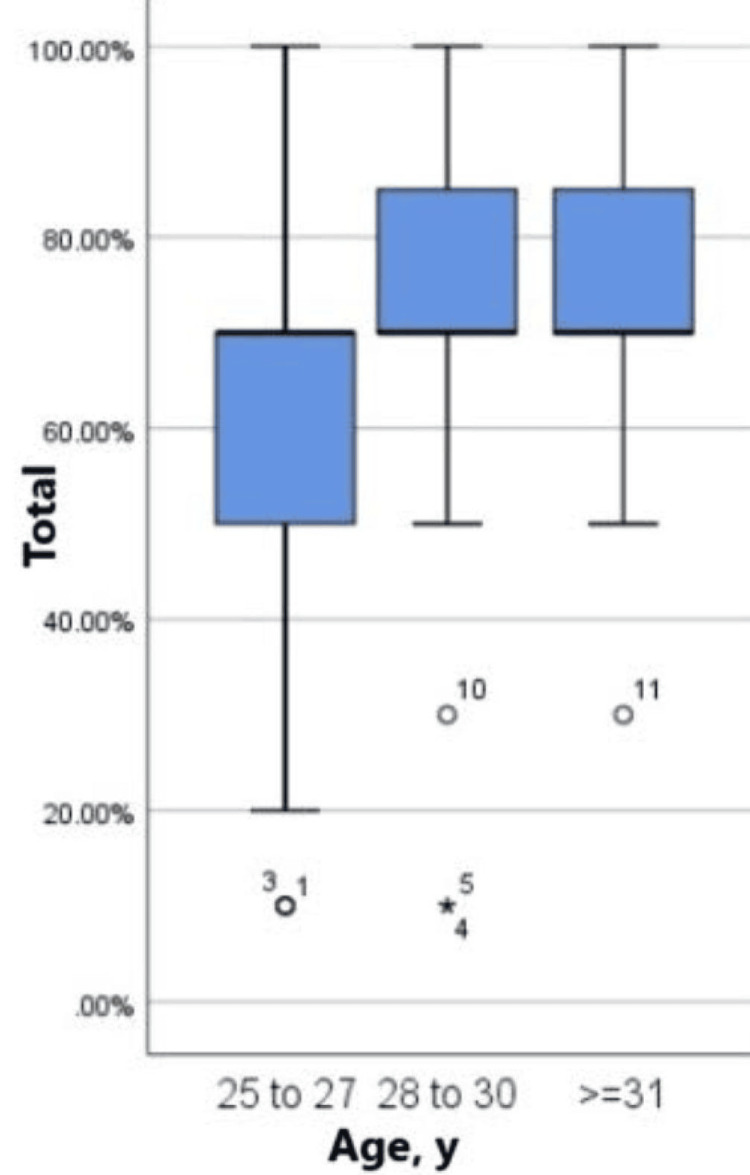
Boxplot of score distribution across different age groups. Age group 25 to 27: The lowest minimum score. The median is close to the third quartile. Data is skewed to the left. There are a few outliers. There is more variation in the scores in comparison to other age groups. Age group 28 to 30: The median is close to the first quartile. Data is skewed to the right. There are a few outliers. Age group ≥31: The median is close to the first quartile. Data is skewed to the right. There are a few outliers.

**Figure 2:**
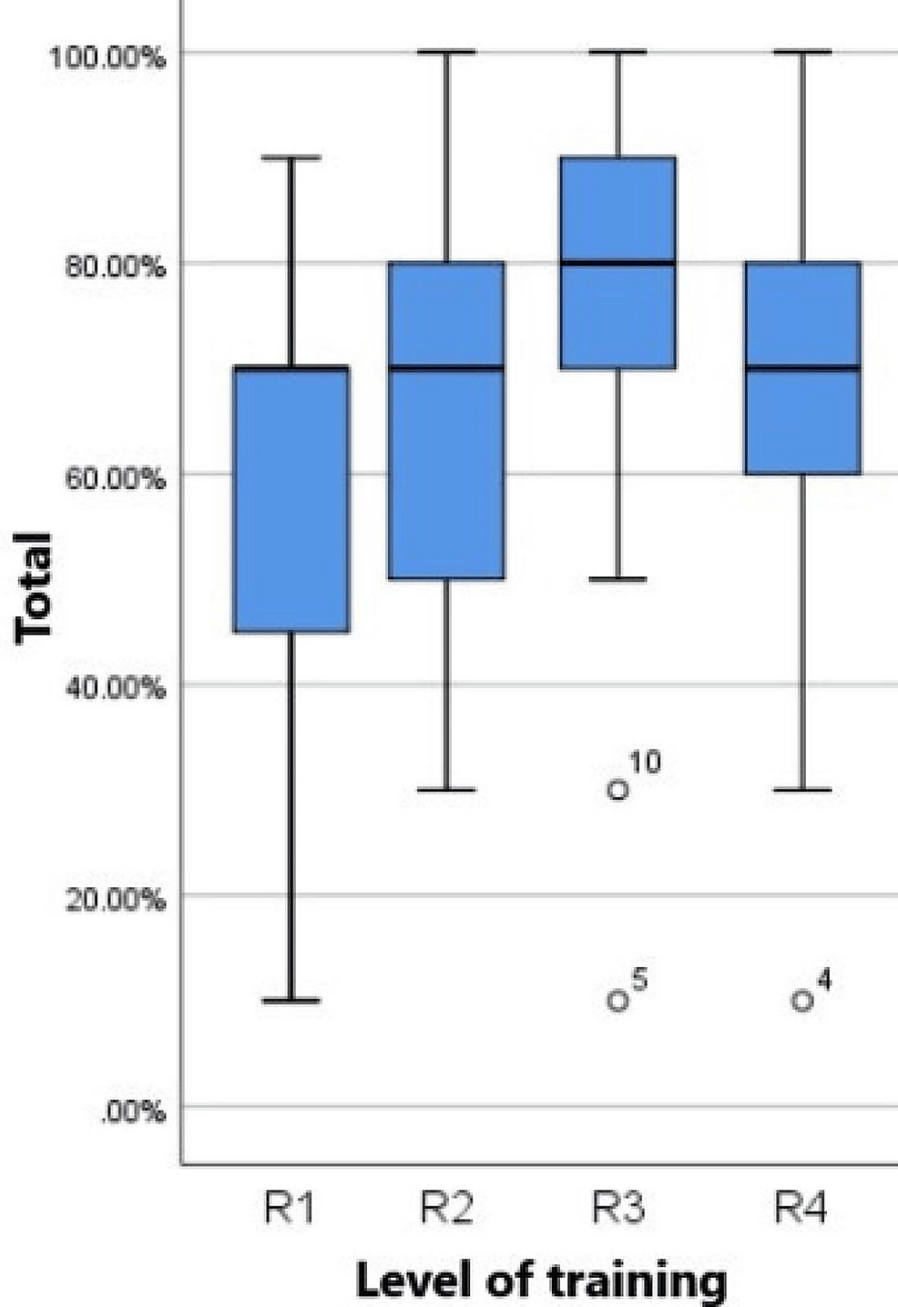
Boxplot of score distribution across different training levels. R1: Data is skewed to the left. There is more variation in the scores in comparison to other groups. R2: The minimum score is 30%. Data is normally distributed. R3: The minimum score for this group is higher than the rest of the groups. There are a few outliers. R4: The minimum score is 30%. Data is normally distributed. R: resident

The distribution of scores across different age groups is illustrated in Figure 1 using a boxplot. The plot demonstrates variation in scores among participants of different age categories, providing insights into performance trends across the age spectrum. Figure 2 depicts the distribution of scores across various training levels, highlighting differences in performance among residents at different stages of training.

**Figure 3:**
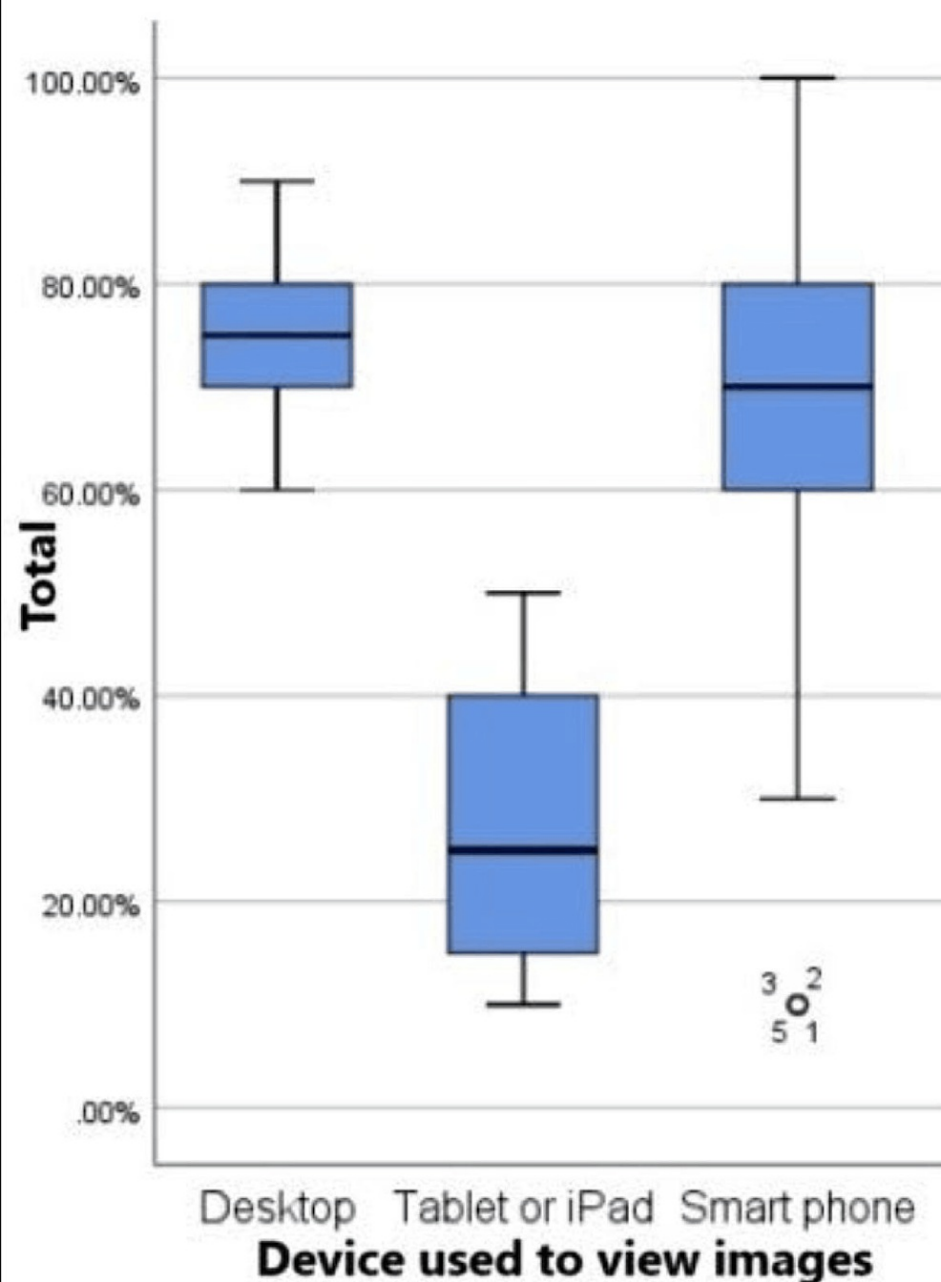
Boxplot of score distribution across devices used to view the X-rays. Desktop: The minimum score is 60% and the maximum is 90% which is the highest in devices. Tablet or iPad: The minimum score is 10% and the maximum is 50% which is the lowest among all devices. Smartphone: The minimum is 30% and the maximum is 100%. There is more variation in scores when using smartphones.

Finally, Figure 3 showcases the distribution of scores based on the device used for viewing X-rays, demonstrating variability in performance associated with the choice of viewing device.

## Discussion

In this study, there was an association between age and interpretation accuracy. Participants aged 28 to 30 years demonstrated the highest mean score, suggesting a potential correlation between experience and competency in interpreting hand X-rays. This finding aligns with previous literature, indicating that residents with more experience tend to exhibit higher levels of accuracy in radiological interpretation [17,18].

Furthermore, our study revealed significant differences in interpretation scores across different training levels. Residents in the third year of training (R3) exhibited the highest mean score, indicating a progressive improvement in competency throughout the residency program. This finding is consistent with previous research, highlighting the positive impact of structured training programs on the development of radiological interpretation skills [19]. However, residents in their fourth year of training performed inferiorly than R3 and showed a lower mean score.

Our findings are consistent with previous studies demonstrating a positive correlation between experience and competency in radiological interpretation [20]. Moreover, it is important to note that age is not a reliable indicator of experience, as factors such as level of supervision, feedback system, exposure to diverse clinical scenarios, and constant teaching and training play a significant role.

Interestingly, our study also identified a significant association between the choice of viewing device and interpretation accuracy. Participants using desktops achieved higher mean scores compared to those using smartphones or tablets. This finding underscores the importance of optimizing viewing platforms to enhance the accuracy and reliability of radiological interpretations. While previous studies have investigated the impact of viewing devices on diagnostic accuracy, further research is needed to elucidate the underlying factors contributing to these differences [21].

Similarly, the observed differences in interpretation scores across training levels corroborate existing literature, highlighting the progressive improvement in radiological interpretation skills throughout residency training [19,20]. Structured educational programs and hands-on experience in interpreting radiological images are essential components of residency training, contributing to the development of diagnostic expertise among residents. Interestingly, the significance of viewing devices in radiological interpretation has been a subject of debate in the literature. While some studies have reported comparable diagnostic accuracy across different viewing platforms [22,23], our findings suggest that the choice of viewing device may impact interpretation accuracy. Further research is warranted to explore the underlying factors contributing to these differences and develop guidelines for optimizing viewing platforms in clinical practice.

Limitations and future directions

Despite the insights gained from our study, several limitations should be acknowledged. First, the use of simulated cases may not fully replicate the complexity of real-life clinical scenarios, potentially affecting the generalizability of our findings. Additionally, the small sample size and the inclusion of participants from a single country limit the external validity of our results, seeing as the training is standardized in the three regions following the same guidelines and teaching methods. Future research incorporating larger and more diverse samples is needed to validate our findings and explore potential cultural and regional differences in radiological interpretation competency.

Furthermore, while our study focused specifically on hand X-rays, future research could investigate interpretation competency in other radiological modalities and clinical contexts. Additionally, longitudinal studies tracking the development of interpretation skills over time could provide valuable insights into the factors influencing competency among EM residents.

## Conclusions

The results of this study suggest that EM residents in Saudi Arabia demonstrate competency in interpreting hand X-rays. The significant associations between age, training level, choice of viewing device, and assessment scores underscore the importance of tailored training programs and optimized technology to enhance diagnostic accuracy and improve patient outcomes in EM settings. Further research could explore additional factors influencing interpretation competency and investigate strategies to further enhance diagnostic proficiency among emergency medicine residents.

## Figures and Tables

**Figure 4:**
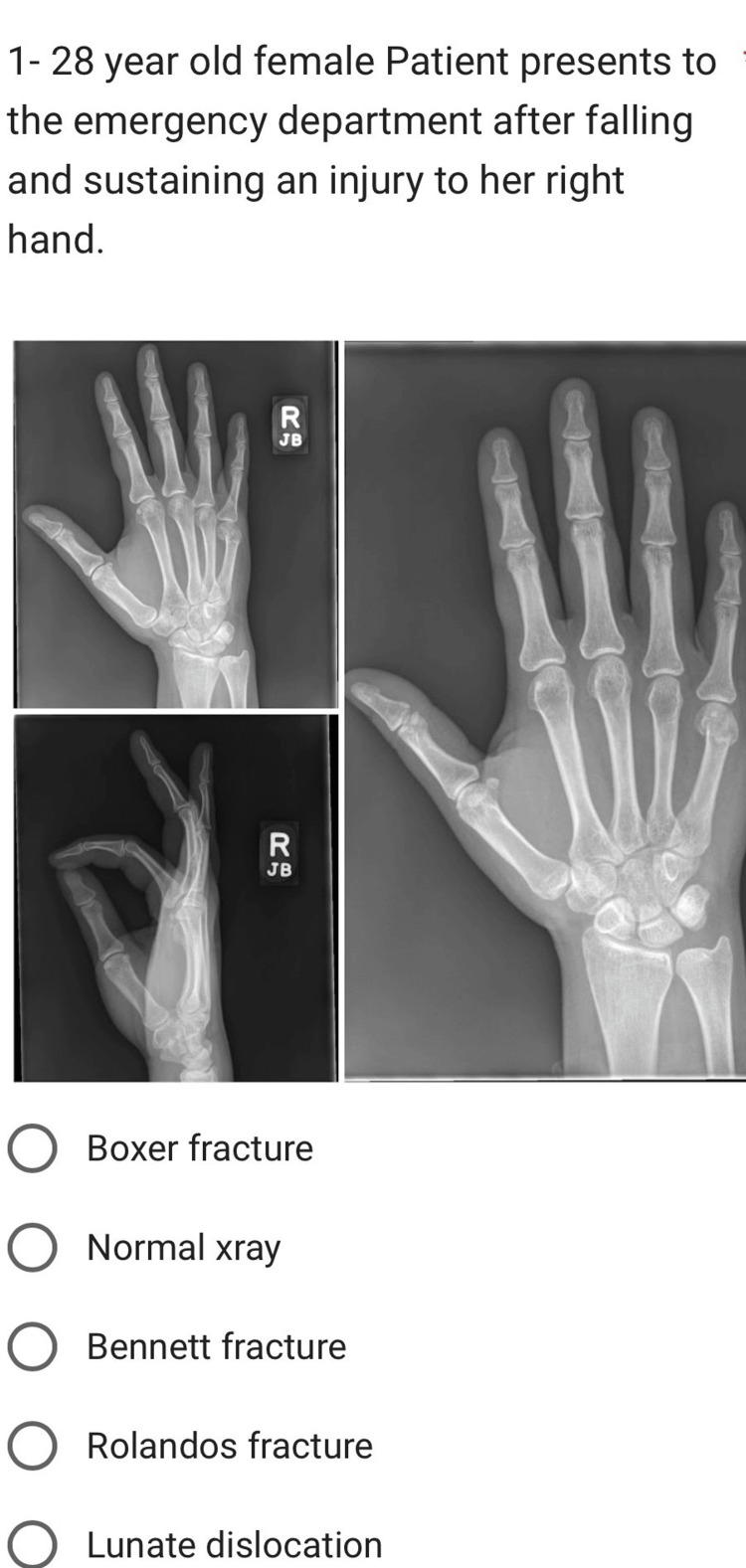
Clinical vignette case 1.

**Figure 5:**
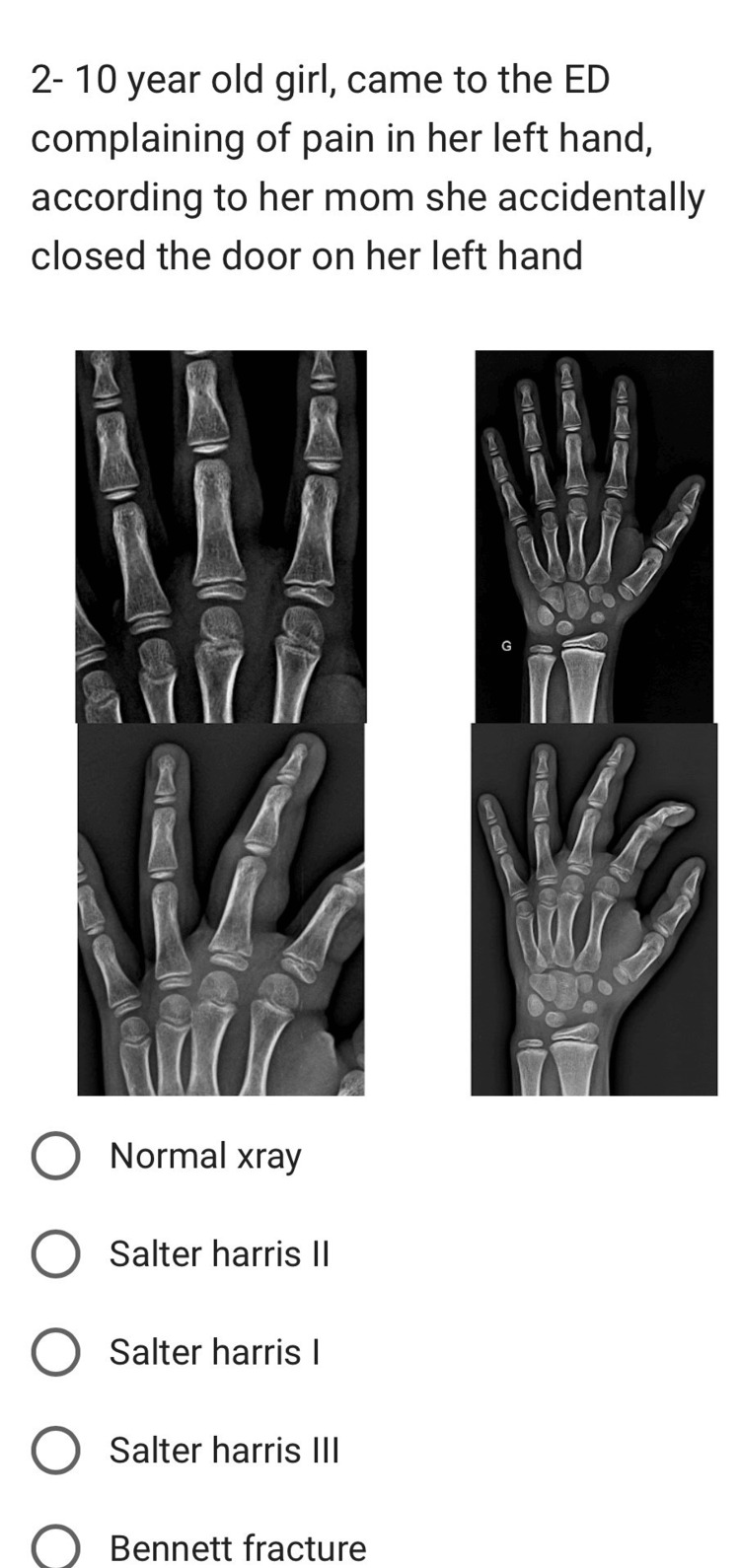
Clinical vignette case 2. ED: emergency department

**Figure 6:**
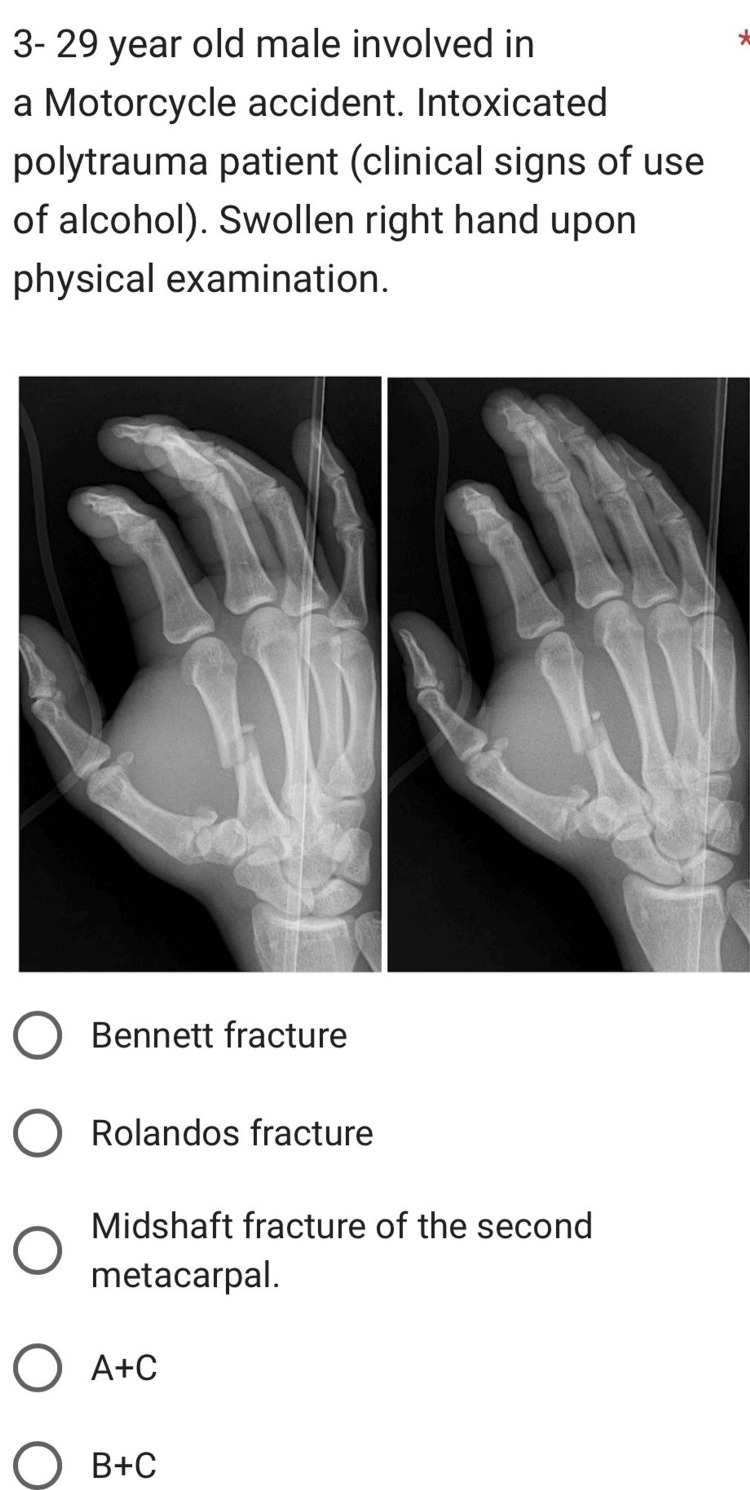
Clinical vignette case 3.

**Figure 7:**
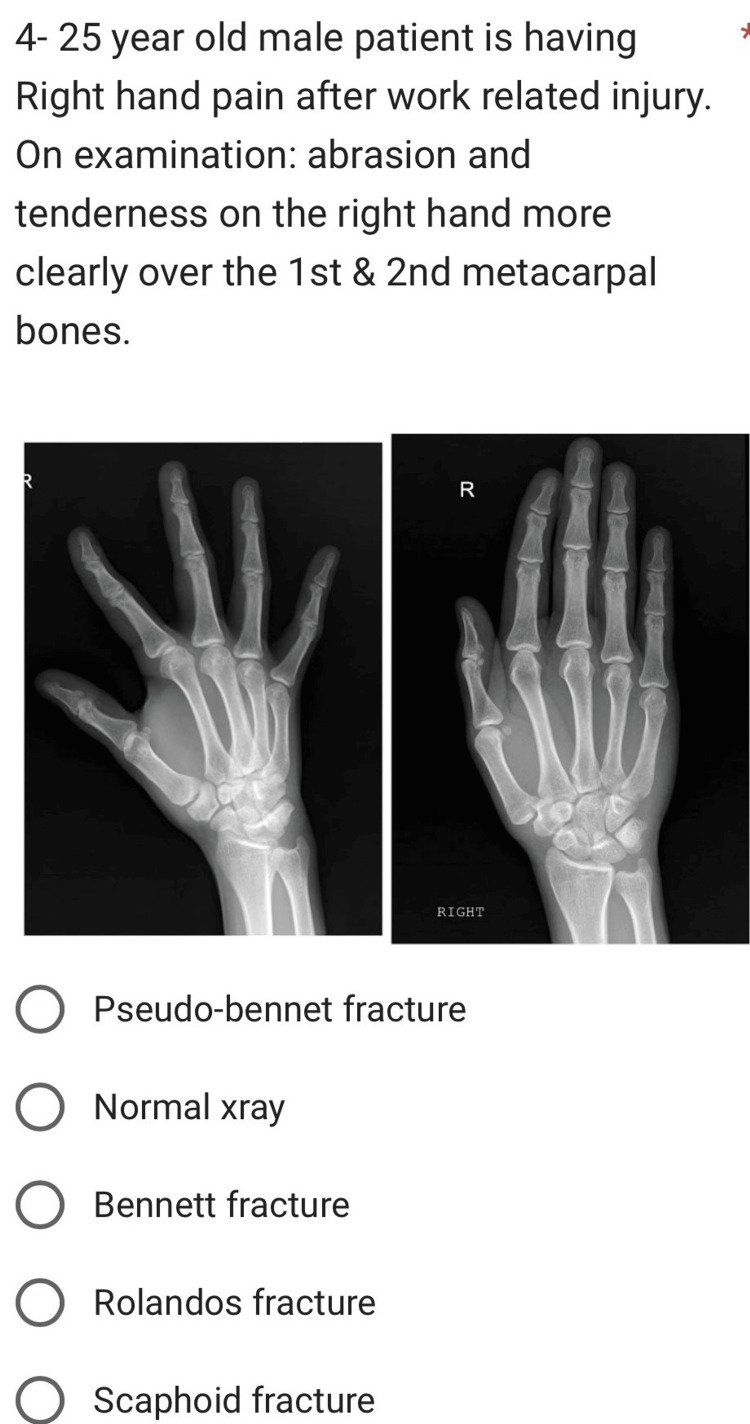
Clinical vignette case 4.

**Figure 8:**
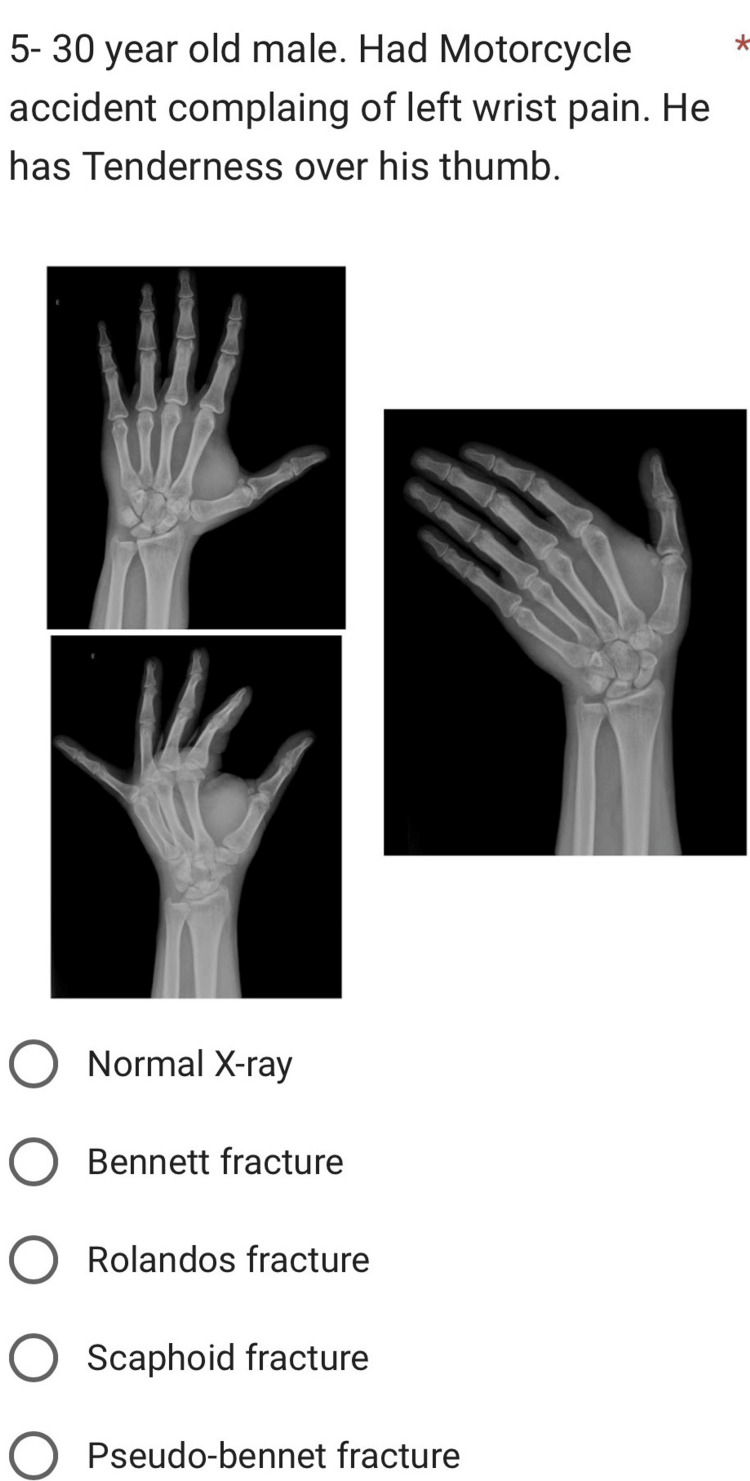
Clinical vignette case 5.

**Figure 9:**
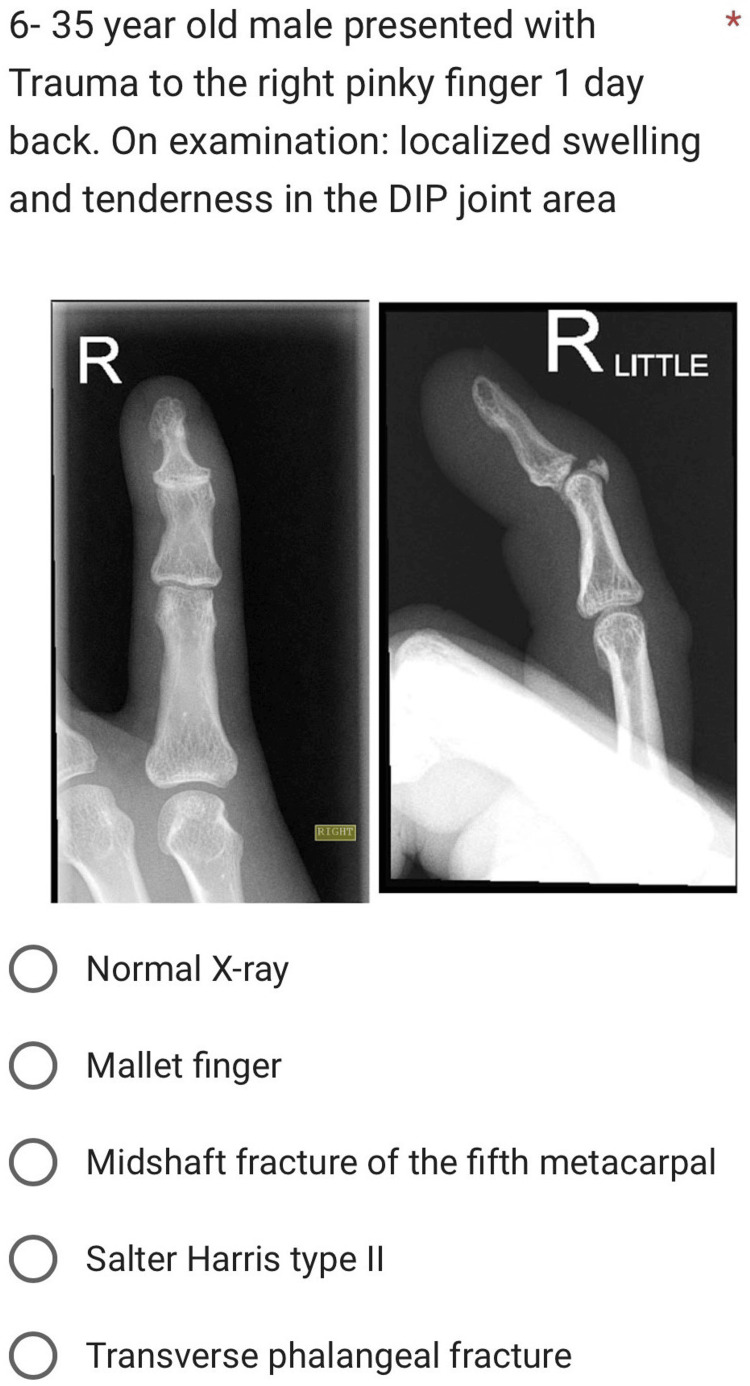
Clinical vignette case 6. DIP: distal interphalangeal joint

**Figure 10:**
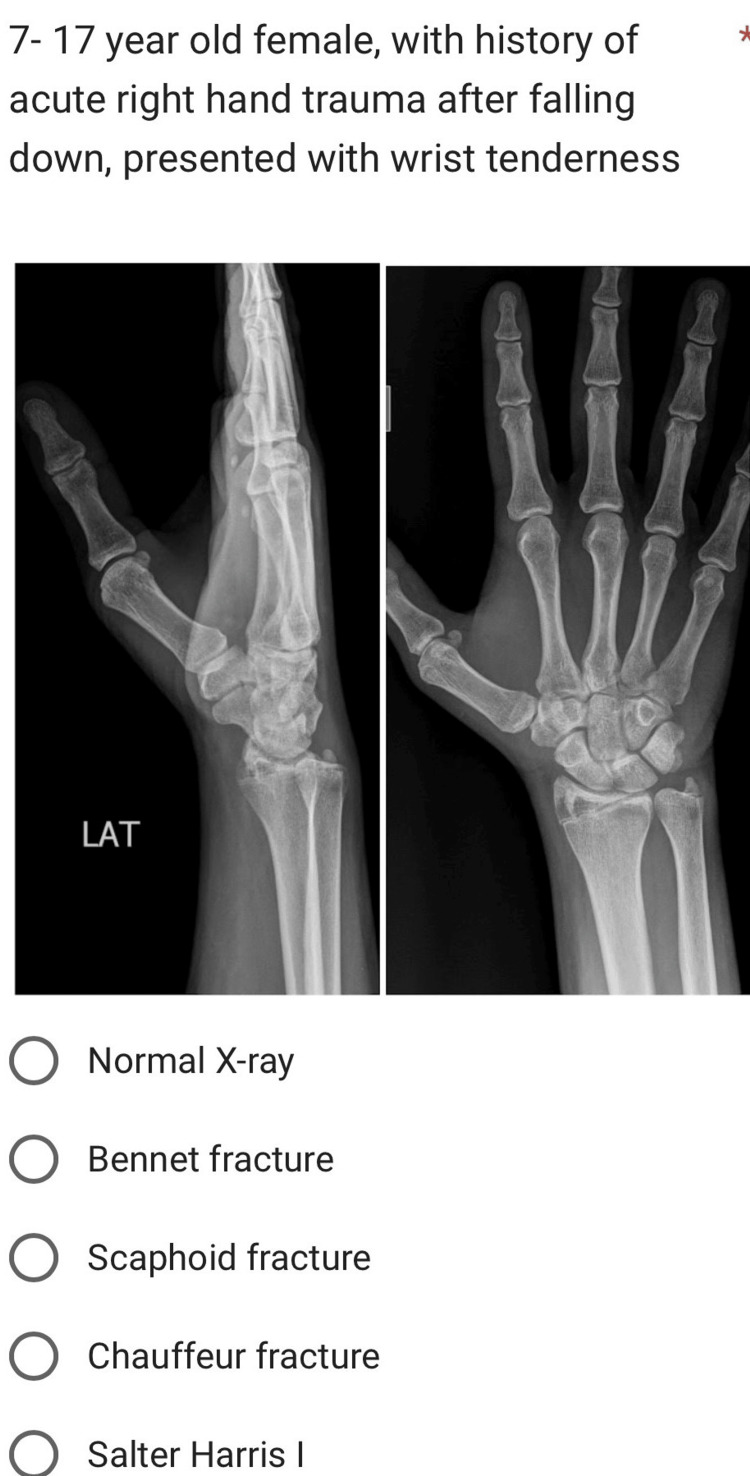
Clinical vignette case 7.

**Figure 11:**
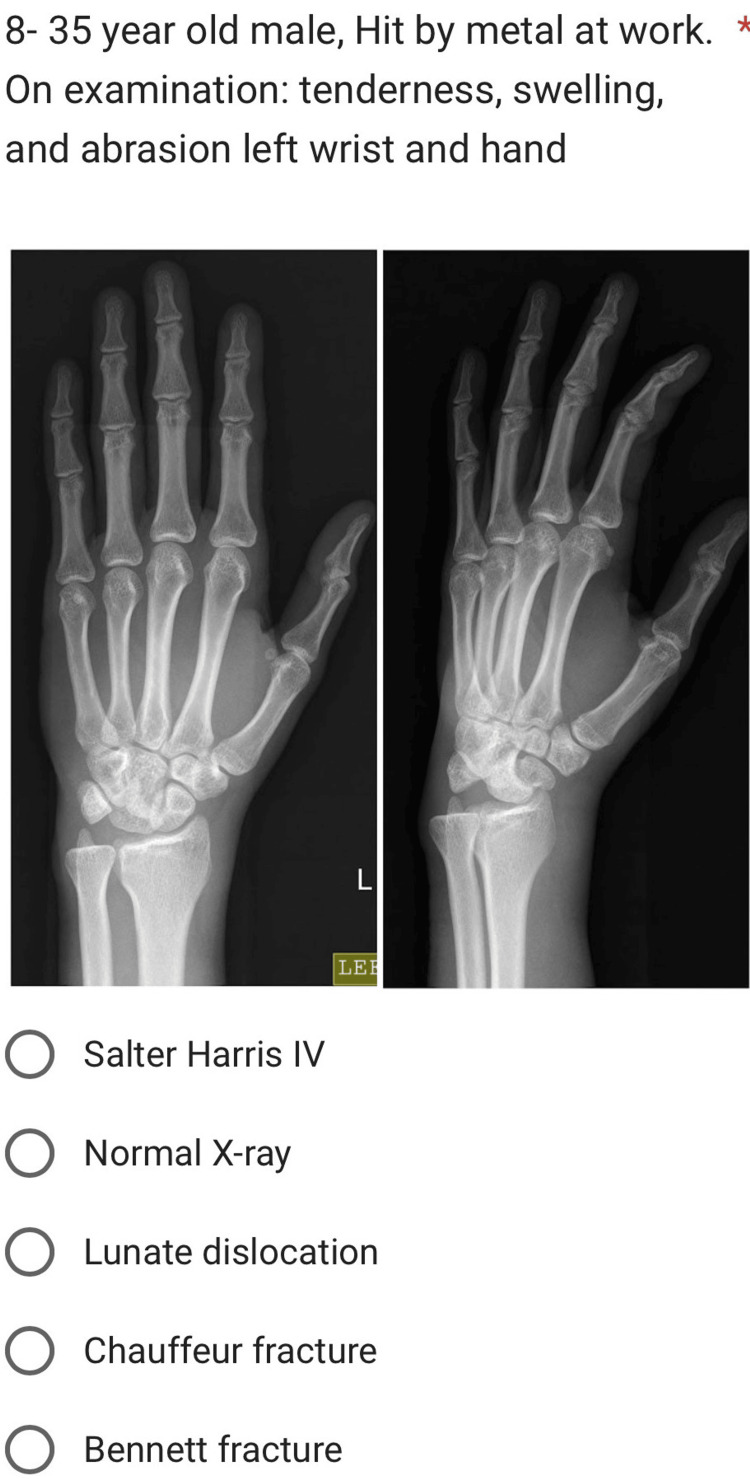
Clinical vignette case 8.

**Figure 12:**
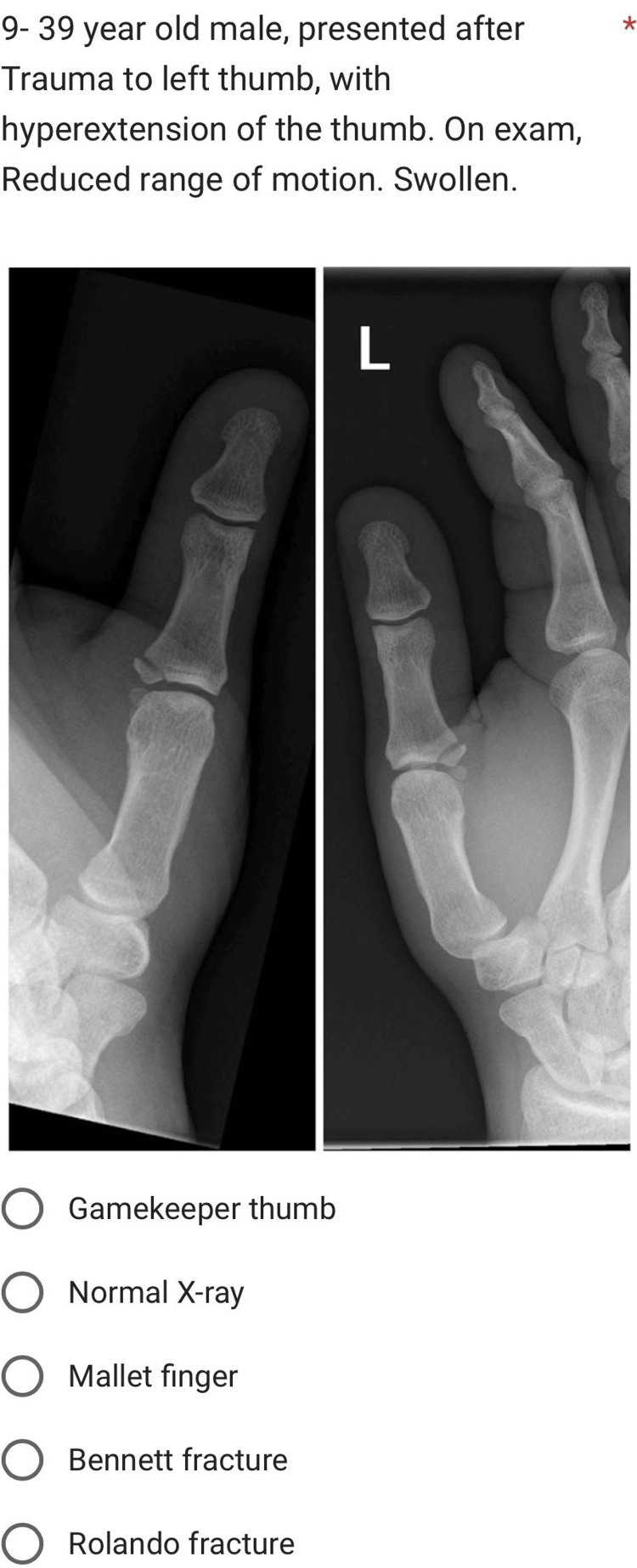
Clinical vignette case 9.

**Figure 13:**
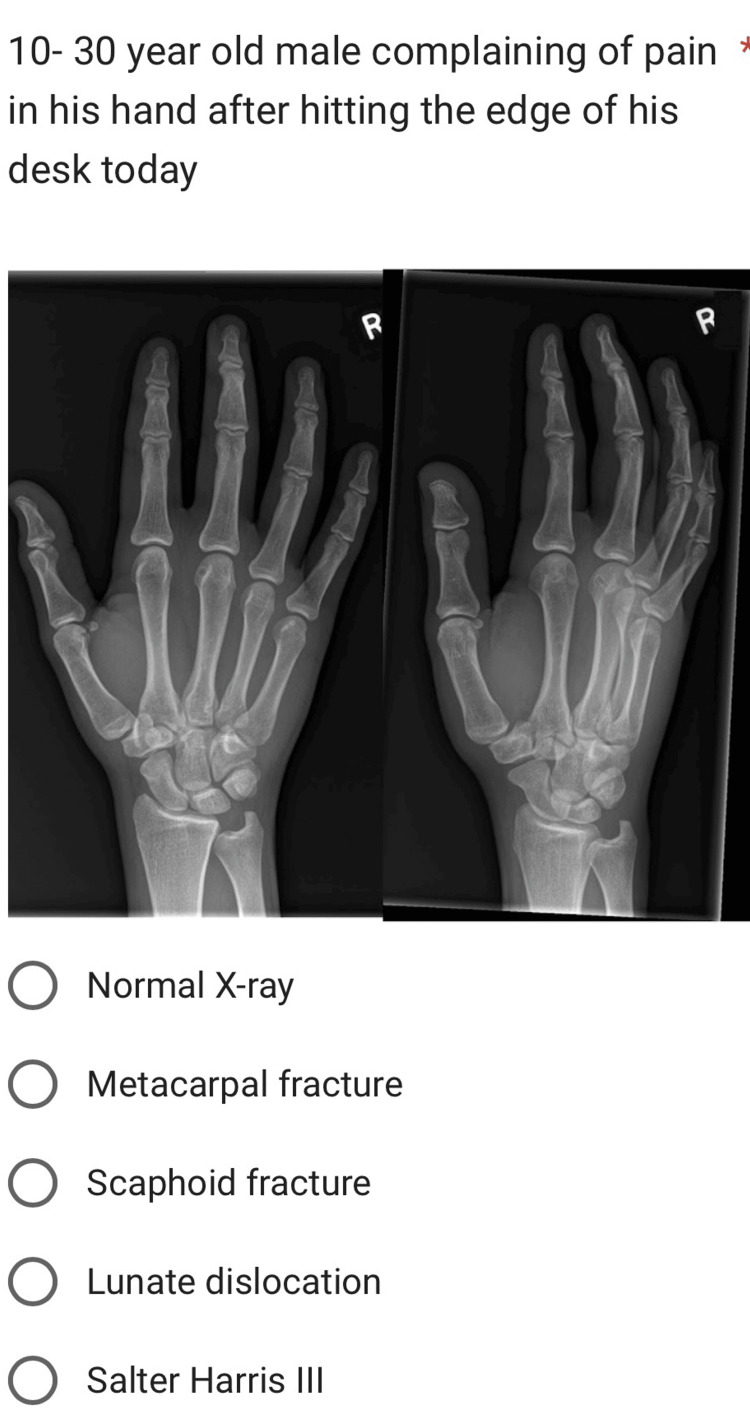
Clinical vignette case 10.
